# P-1178. Trends in invasive methicillin-resistant *Staphylococcus aureus* infections among children in the United States, 2011–2021

**DOI:** 10.1093/ofid/ofae631.1364

**Published:** 2025-01-29

**Authors:** Mohsin Ali, Jessica M Healy, Kelly A Jackson, Joelle Nadle, Susan Petit, Susan M Ray, Ruth Lynfield, Carmen Bernu, Lee Harrison, Ghinwa Dumyati, Marissa Walsh, Tiffanie M Markus, William Schaffner, Isaac See, Holly M Biggs

**Affiliations:** CDC, Decatur, Georgia; Centers for Disease Control and Prevention, Atlanta, Georgia; U.S. Centers for Disease Control and Prevention, Atlanta, Georgia; California Emerging Infections Program, Oakland, California; Connecticut Department of Public Health, Hartford, Connecticut; Emory University School of Medicine, Atlanta, Georgia; Minnesota Department of Health, St. Paul, MN; Minnesota Department of Health, St. Paul, MN; University of Pittsburgh, Pittsburgh, PA; New York Emerging Infections Program and University of Rochester Medical Center, Rochester, New York; New York Emerging Infections Program, Rochester, New York; Vanderbilt University Medical Center, Nashville, Tennessee; Vanderbilt University Medical Center, Nashville, Tennessee; U.S. Centers for Disease Control and Prevention, Atlanta, Georgia; U.S. Centers for Disease Control and Prevention, Atlanta, Georgia

## Abstract

**Background:**

During 2005–2010, incidence of community-associated (CA) invasive methicillin-resistant *Staphylococcus aureus* (MRSA) infections among children was rising, whereas hospital-onset (HO) rates were unchanged except for a decline among young infants. Recent trends in invasive MRSA infections among children have not been described.

**Figure 1.**
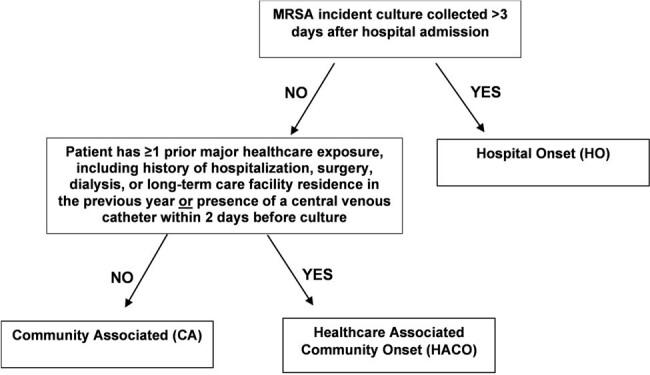
Epidemiologic classification of cases

**Methods:**

We analyzed invasive MRSA cases in children aged < 18 years reported during 2011–2021 through active population- and laboratory-based surveillance by CDC's Emerging Infections Program in seven U.S. metropolitan areas. A case was defined as isolation of MRSA from a normally sterile body site in a surveillance area resident. Cases aged ≥3 days were assigned an epidemiologic class based on healthcare exposure: HO, healthcare-associated community-onset (HACO), or CA (Figure 1). Annual incidence was calculated using U.S. census population estimates and stratified by age category and epidemiologic class. Crude incidence-rate ratios (IRR) were modeled using negative-binomial regression.

**Table 1.**
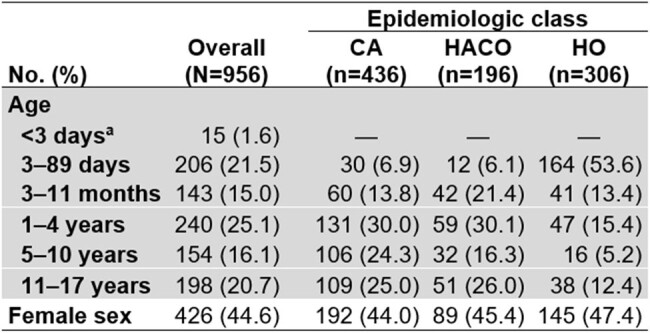
Demographic characteristics of children with invasive methicillin-resistant Staphylococcus aureus (MRSA) infections overall and by epidemiologic class, Emerging Infections Program, seven U.S. metropolitan areas, 2011–2021 Abbreviations: CA, community-associated; HACO, healthcare-associated community-onset; HO, hospital-onset. a: Children aged <3 days were not assigned an epidemiologic class

**Results:**

Of 956 cases reported, 938 (98%) were assigned an epidemiologic class: 436 (47%) CA, 197 (21%) HACO, and 306 (33%) HO. Infants (3 days–11 months) accounted for 21% of CA, 28% of HACO, and 67% of HO cases (Table 1). Overall incidence declined by 6% per year on average, from 3.7 per 100,000 population in 2011 to 2.1 in 2021 (Table 2 and Figure 2). Incidence of CA and HACO cases declined, whereas HO rates did not significantly change. Compared to 11–17-year-olds, overall incidence was highest (30-fold greater) among 3–89-day-olds (Table 2); 164 (80%) cases in this age group were HO. Nearly all (152, 93%) HO cases among 3–89-day-olds had bloodstream infection (BSI); among 47 cases during 2019–2021 with additional clinical data, 46 (98%) were admitted to the neonatal intensive care unit (NICU) at birth, 39 (83%) were born premature, and 29 (62%) had a central venous catheter within two days prior to MRSA culture.

**Table 2.**
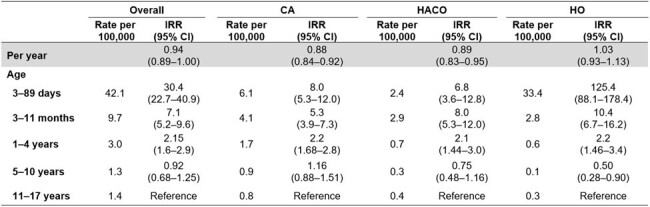
Incidence of invasive methicillin-resistant Staphylococcus aureus (MRSA) infection among children per year and by age category, overall and stratified by epidemiologic class, Emerging Infections Program, seven U.S. metropolitan areas, 2011–2021. Abbreviations: CA, community-associated; CI, confidence interval; HACO, healthcare-associated community-onset; HO, hospital-onset; IRR, incidence-rate ratio.

**Conclusion:**

During the past decade, nearly half of invasive MRSA infections among children were CA. Incidence of CA and HACO cases, primarily occurring among children aged >1 year, declined. However, HO rates remained disproportionately high among young infants, emphasizing the importance of BSI prevention among infants in the NICU.

**Figure 2.**
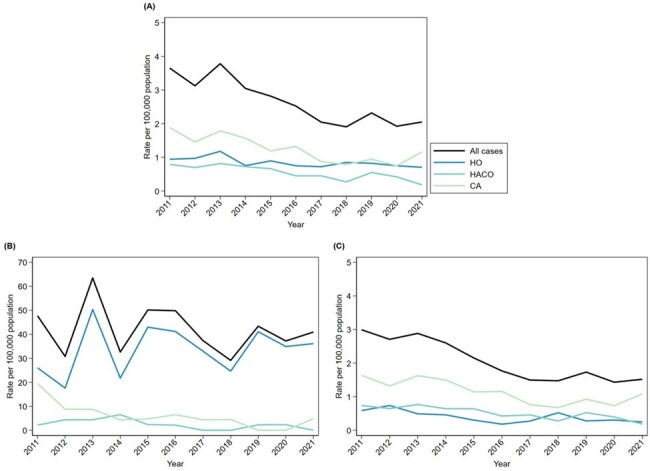
Crude annual incidence of invasive methicillin-resistant Staphylococcus aureus (MRSA) infections, overall and by epidemiologic class, for (A) all children, (B) children aged 3–89 days, and (C) children aged 90 days–17 years, Emerging Infections Program, seven U.S. metropolitan areas, 2011–2021.

**Disclosures:**

**Lee Harrison, MD**, GSK: Advisor/Consultant|Merck: Advisor/Consultant|Pfizer: Advisor/Consultant|Sanofi: Advisor/Consultant

